# Londinensis in Reply to Mr. Smerdon

**Published:** 1815-07

**Authors:** 

**Affiliations:** West Smithfield


					For the Medical and Physical Journal.
Londinensis in Reply to Mr. Smerdon.
I
HAVE to thank Mr. Smerdon for his answers to some
queries of mine, inserted in No. 198 of your Journal;
but, as, at the conclusion of those answers, he has had the
candour to acknowledge, that not any thing which he has
written amounts to more than an hypothesis, and, of conse-
quence, being unable to adduce any evidence, either for the
existence of a principle of irritability, or that of a nervous
fluid, I have nothing further to say: " -hypotheses, vel meta-
*o. 197. J> -physical
13 Dr. James Jackson pn Inflanwation of iht
m QC?i4i<v:umf
nQii habent
pfiysicte, ypl phyiiq#, vel qwliiatum Qcculfar-Wm, $eu m$i
dwniccE) in philosophic [mea] locum nq
LONDIN;ENSIS,
West Smithjidd;
Jme%) J8la.

				

